# A role for neutrophils in early enthesitis in spondyloarthritis

**DOI:** 10.1186/s13075-021-02693-7

**Published:** 2022-01-17

**Authors:** Zheni Stavre, Charles Bridgewood, Qiao Zhou, Yukiko Maeda, Ting-ting Huang, Jozsef Karman, Almas Khan, Sami Giryes, Kassem Sharif, Dennis McGonagle, Ellen M. Gravallese

**Affiliations:** 1 Department of Medicine/Division of Rheumatology, University of Massachusetts Chan Medical School, Worcester, MA 01605 USA; 2 The Leeds Institute of Molecular Medicine, University of Leeds, Leeds, UK; 3 Sichuan Academy of Medical Sciences & Sichuan Provincial People’s Hospital, University of Electronic Science and Technology of China, Chengdu, Sichuan Province China; 4 Abbvie Cambridge Research Center, Cambridge, MA 02139 USA; 5 Leeds Teaching Hospitals NHS Trust, Leeds, UK; 6 Leeds Institute of Rheumatic and Musculoskeletal Medicine (LIRMM), University of Leeds, Leeds, UK; 7 Sheba Medical Center, Tel Aviv, Sackler Faculty of Medicine, Tel Aviv University, Tel Aviv, Israel; 8 Department of Medicine/Division of Rheumatology, Inflammation and Immunity, Brigham and Women’s Hospital, Boston, MA 02115 USA

**Keywords:** Spondyloarthritis, Neutrophils, Enthesis

## Abstract

**Background:**

Neutrophils are present in the early phases of spondyloarthritis-related uveitis, skin and intestinal disease, but their role in enthesitis, a cardinal musculoskeletal lesion in spondyloarthritis, remains unknown. We considered the role of neutrophils in the experimental SKG mouse model of SpA and in human axial entheses.

**Methods:**

Early inflammatory infiltrates in the axial and peripheral entheseal sites in SKG mice were evaluated using immunohistochemistry and laser capture microdissection of entheseal tissue. Whole transcriptome analysis was carried out using Affymetrix gene array MTA 1.0, and data was analyzed via IPA. We further isolated neutrophils from human peri-entheseal bone and fibroblasts from entheseal soft tissue obtained from the axial skeleton of healthy patients and determined the response of these cells to fungal adjuvant.

**Results:**

Following fungal adjuvant administration, early axial and peripheral inflammation in SKG mice was characterized by prominent neutrophilic entheseal inflammation. Expression of transcripts arising from neutrophils include abundant mRNA for the alarmins S100A8 and S100A9. In normal human axial entheses, neutrophils were present in the peri-entheseal bone. Upon fungal stimulation in vitro, human neutrophils produced IL-23 protein, while isolated human entheseal fibroblasts produced chemokines, including IL-8, important in the recruitment of neutrophils.

**Conclusion:**

Neutrophils with inducible IL-23 production are present in uninflamed human entheseal sites, and neutrophils are prominent in early murine spondyloarthritis-related enthesitis. We propose a role for neutrophils in the early development of enthesitis.

**Supplementary Information:**

The online version contains supplementary material available at 10.1186/s13075-021-02693-7.

## Introduction

Spondyloarthritis (SpA) includes a family of diseases that share a common genetic predisposition and several clinical manifestations. These diseases include ankylosing spondylitis (AS), reactive arthritis (ReA), psoriatic arthritis (PsA) and inflammatory bowel disease (IBD)-related arthritis. The enthesis represents the insertion point where tendons or ligaments attach to the bone and inflammation of this anatomic site (enthesitis) is a cardinal feature of SpA.

Neutrophils are innate immune cells implicated in the pathogenesis of psoriasis, PsA, uveitis and IBD [1–5]. They are prominent in other diseases that also fall within the SpA spectrum, including synovitis, acne, pustulosis, hyperostosis and osteitis (SAPHO) and chronic recurrent multifocal osteomyelitis (CRMO) [6, 7]. Neutrophils are also present in the sacroiliac joints of patients with early sacroiliitis and suspected AS [8]. Moreover, diseases in the SpA family share many common genes within the IL-17/IL-23 axis, a cytokine axis central to neutrophil immunobiology [9–15]. Although neutrophils are the most abundant leukocytes in the circulation and are a first line of innate immune defense, they are difficult to study because they are short-lived and, following production in the bone marrow, enter the circulation, migrate into tissues and then are rapidly cleared by macrophages [16]. Considering the evidence of their presence in SpA-related inflammatory conditions, we investigated early entheseal inflammation and tested the response of neutrophils to fungal adjuvants that are known to initiate enthesitis in murine models.

The SKG mouse model has emerged as a useful tool for studying SpA. Arthritis in these mice can occur spontaneously but is accelerated and coordinated by exposure to fungal or bacterial cell wall components such as zymosan, curdlan or mannan [17]. While initially described as a model of rheumatoid arthritis, more recent evidence demonstrates that SKG mice have many features of the SpA spectrum of disease, including spondylitis, dactylitis, enteritis and psoriasiform skin lesions [18–20]. Disease in this model is attributed to increased T cell autoreactivity due to a mutation in Zeta chain Associated Protein kinase 70 (ZAP-70). ZAP-70 is a component of the T cell receptor complex, and this mutation alters the T cell repertoire, resulting in an increase in Th1 and Th17 cells. In addition, neutrophils and the CARD9 signaling axis are essential components of arthritis induction in this model [21].

Tissue infiltrating neutrophils have been reported to produce pro-inflammatory cytokines and have been proposed as the main source of IL-23 in the colonic tissue from IBD patients [22]. Within SpA and the wider field of inflammation, understanding the cellular sources of IL-23 is an active area of research. While professional antigen presenting cells such as macrophages and dendritic cells are major sources of IL-23, other potential sources including epithelial cells have been identified [23]. The production of IL-23 by neutrophils was recently shown in in vitro studies [24]. Given the close association between IBD and SpA, we investigated the potential role of neutrophils in human entheses and in the axial and peripheral entheseal tissues in the murine SKG model. We demonstrate a strong representation of neutrophils in the early inflammatory infiltrate at axial and peripheral entheseal sites in SKG mice. We also confirm the presence of neutrophils at the normal human spinal enthesis and show that human entheseal neutrophils have the ability to secrete IL-23. We therefore hypothesize that neutrophils may be important players in the innate immune response in the early phase of enthesitis in SpA.

## Materials and methods

### The SKG mouse model

All animal procedures were performed in accordance with protocols approved by the Institutional Animal Care and Use Committee at the University of Massachusetts Chan Medical School. ZAP-70W163C mutant BALB/c (SKG) mice were kindly gifted by Dr. Shimon Sakaguchi [25]. Female SKG mice and control female BALB/c mice (*n* = 6 SKG, *n* = 4 BALB/c) were administered curdlan, 3mg (Wako Chemicals), by a single intraperitoneal injection at 9 to 10 weeks of age. Peripheral joint inflammation was scored weekly using the clinical criteria described previously [19]. Body weight and caliper measurements for ankle widths were also recorded weekly. Mice were euthanized at serial time points up to 11 weeks post curdlan injection and spines and hind limbs were collected, fixed in 4% paraformaldehyde for 48 h, transferred to 70% ethanol and stored at 4°C.

### Procurement of murine-inflamed entheseal tissue via laser capture microscopy

Formalin-fixed, paraffin-embedded (FFPE) tissue samples were obtained from entheseal sites around the ankle and spine vertebrae 3 weeks after curdlan injection and subjected to laser capture microscopy (*n* = 3 SKG, *n* = 3 BALB/c control). The 8-μm sections were cut and mounted on membrane slides (MMI) in RNase-free conditions, deparaffinized in 3 changes of xylene and allowed to air dry under vacuum prior to laser capture microdissection (LCM). In addition, one 4-μm section between every 3 membrane slide sections was cut onto a glass slide and stained with hematoxylin and eosin (H&E) using a Leica Autostainer XL. Three membrane slides were mounted on an Olympus IX81 microscope. The corresponding H&E section was placed on an Olympus BX41 microscope to visualize entheseal inflammation sites. LCM was performed on 8-μm sections using an MMI Cell Cut laser capture microdissection system (MMI Inc., Rockledge, FL). Using MMI Cell Tools software (Version celltools-4.4 #261), areas to be microdissected with the laser were circumscribed on a monitor using the MMI system stylus. Multiple serial sections were required to ensure a minimum yield of 50ng of RNA. Microdissected entheses were collected onto MMI caps, pooled and subsequently incubated in 150μl of digestion buffer containing 10μl of proteinase K (miRNeasey FFPE Kit, QIAGEN) at 55^o^C overnight and stored at −80^o^C until all tissue samples were collected. 50ng of total RNA was amplified using the SensationPlus FFPE amplification kit and subjected to whole transcriptome analysis using Affymetrix gene array MTA 1.0.

### Immunohistochemistry

Immunohistochemical staining was performed on 5-μm FFPE vertebral or ankle sections that were dried for a minimum of 48 h at 37^o^C using a modification of a published protocol [26]. Slides were deparaffinized by transferring from xylene through graded ethanol concentrations. Epitope retrieval was performed in a microwave oven with citrate buffer. Endogenous peroxidase was inhibited by incubation in 3% H_2_O_2_, followed by avidin/biotin blocking (Invitrogen, Camarillo, CA). Primary rat anti-mouse S100A8 antibody (clone MRP-8, MyBioSource, San Diego, CA) was used at a 1:1000 dilution. Control immunostaining was performed on parallel sections from each group with an isotype rat IgG2b antibody (AbD Serotec, Puchheim, Germany). Sections were stained with a biotinylated secondary donkey anti-rat antibody (Jackson ImmunoResearch, West Grove, PA) diluted 1:750, followed by detection with SA-HRP (Dako, Glostrup, Denmark) and diaminobenzidine (DAB) (Dako, Glostrup, Denmark). MPO staining of murine slides was performed by ServiceBio Inc. (Boston, MA) using a polyclonal rabbit anti-mouse MPO antibody (primary, ServiceBio Inc. Cat# GB11224) at 1mg/ml storage concentration and a secondary HRP-labelled goat anti-rabbit antibody diluted to 1:200 in PBS. Nuclei were counterstained with hematoxylin. Control immunostaining was performed on parallel sections with rabbit (DA1E) mAb IgG XP isotype control (Cell Signaling Technology, Danvers, MA). Images of MPO-stained sections were obtained by ServiceBio Inc. via brightfield scanning and viewed with QuPath software [27].

### Procurement of the human entheseal tissue

Spinous process entheseal tissue was obtained from 10 patients (5 men and 5 women, median age of 46 years) undergoing spinal decompression, or surgery for scoliosis correction of the thoracic or lumbar spine, using previously described methods [28]. Three patient samples were used for histology, 4 samples were used for IL-23 induction and 3 samples were used for neutrophil and fibroblast isolation. All samples were collected following informed written consent with relevant ethical approval. The study protocol was approved by the North West-Greater Manchester West Research Ethics Committee.

Samples were divided into peri-entheseal bone and entheseal soft tissue. Sections of bone or entheseal soft tissue were digested with collagenase as previously described [28]. Following digestion, cells were filtered, and red cells lysis was performed using ammonium chloride.

### Histology of human entheses

Human enthesis samples (*n*=3) were fixed in 4% paraformaldehyde in a 0.1 M phosphate buffer before decalcifying in EDTA (0.5M) at 4°C. The enthesis tissue was then fixed for 24 h in formalin and mounted in paraffin blocks for histological applications. Tissue sections (5 μm) were deparaffinized in xylene and subsequently rehydrated with graded ethanol series to water. Sections were stained with rabbit anti-human Myeloperoxidase (MPO) (1:50) (Abcam) and EnVision Plus horseradish peroxidase stain (Dako) and counterstained with Mayer’s hematoxylin. Slides were then scanned on Leica Aperio AT2 up to an original magnification 20x and images were captured by using an Aperio Imagescope at a digital magnification of 10x.

### Isolation of human entheseal neutrophils and cell culture

To confirm the presence of neutrophils in the human entheseal tissue, cells released following enthesis digestion (*n*=3), described above, were incubated with 10% mouse serum and 1% IgG in FACS buffer prior to incubation with antibodies. Cells were then stained with DAPI (live/dead), CD45+ (leukocytes), CD66b+ (granulocytes) and CD16+ (neutrophils). Cells were analyzed using the FACSMELODY (BD Biosciences) and FlowJo software (Tree Star Software, San Carlos, California, USA). All lists of antibodies, clones and manufacturers can be found in the supplementary material (Supplementary Table 1). For functional studies, neutrophils were isolated from digested entheseal tissue using a neutrophil isolation kit (STEMCELL Technologies, Cambridge, MA). This kit was validated for neutrophil purity prior to proceeding with fungal adjuvant stimulation. After 48 h of culture, isolated neutrophils evaluated through flow cytometry show 94% purity for CD66b. Isolated entheseal neutrophils were subsequently cultured in RPMI (Sigma-Aldrich) supplemented with 10% FCS and 1% penicillin/streptomycin (Invitrogen). 5x10^5^ neutrophils were plated in 96-well plates and stimulated with Zymosan (0.5mg/ml) (Sigma-Aldrich), IFNγ (20 ng/ml) (Peprotech) or Zymosan plus IFNγ for 48 h (*n*=4). The supernatant was then probed for IL-23 secretion by ELISA (ThermoFisher) according to the manufacturer’s instructions.

### Isolation and stimulation of human entheseal fibroblasts

Entheseal fibroblasts were isolated by digestion of enthesis soft tissue samples as previously described [29]. Cells released following enthesis digestion (*n*=3) were plated in T75 flasks in DMEM media (Gibco, Life Technologies) containing 10% FBS and 1% penicillin/streptomycin. Nonadherent cells were removed with media changes and entheseal fibroblasts were allowed to reach confluency. Following passaging, entheseal fibroblasts were plated in 6 well plates and allowed to reach confluency. Entheseal fibroblasts were tested for CD90 expression to confirm lineage and purity (data not shown). Entheseal fibroblasts were then stimulated with mannan (1 mg/ml). Culture supernatant was probed by ELISA for CCL2, IL-6, IL-8 (Thermofisher) and CCL20 (Biolegend). All ELISA assays were carried out according to the manufacturers’ instructions. Entheseal fibroblasts were also analyzed for the surface expression of an array of adhesion molecules. Entheseal cells were detached with trypsin and blocked with 10% mouse serum and 1% IgG in FACS buffer prior to incubation with antibodies. Cells were stained with mouse anti-human CD54/ICAM-1 and CD106/VCAM-1. Cells were analyzed using LSRII (BD Biosciences) and FlowJo software. Antibodies used are summarized in Supplementary Table 1.

### Statistics

Statistical analysis was conducted using GraphPad Prism software (La Jolla, CA, USA). Paired *t* tests were used to calculate significance. Specific statistical tests are described in the corresponding figure legends. Error bars represent standard deviation. Significant differences between control and test groups were evaluated with *p* values less than *p < 0.05 and **p < 0.01, as indicated.

Signaling Pathway Impact Analysis (SPIA) was performed using the R implementation of the SPIA method [30]. Gene signatures were calculated using the GSVA method implemented as an R package [31] and a previously published signature of resting granulocytes from human peripheral blood [32].

## Results

### Prominent neutrophil infiltration is present at axial and peripheral entheseal sites at early timepoints in SKG mice

**Fig. 1:**
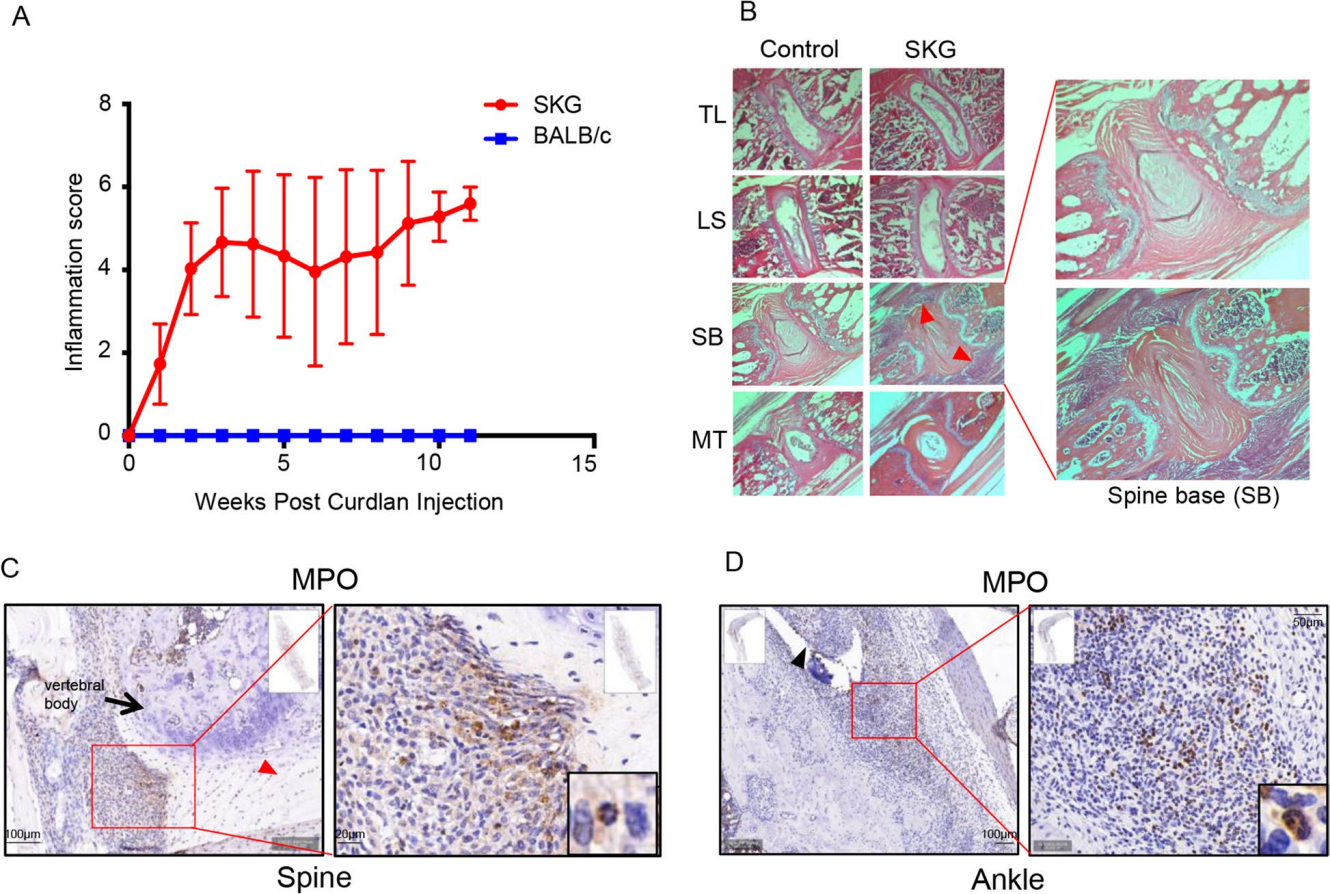
Early inflammatory infiltrates at entheses show abundant neutrophils. **A** Clinical peripheral inflammation scores recorded weekly over a period of 11 weeks post intraperitoneal curdlan injection (*n* = 6 SKG, *n* = 4 BALB/c controls). **B** Prominent inflammatory infiltrates (red arrowheads) in female SKG mice (6 weeks post curdlan injection) are seen in H&E-stained sections (×4 magnification) at the spine base (SB), a region of high mobility. Lesser infiltrates are present in other regions of the spine, including the thoracolumbar (TL), lumbosacral (LS) and mid tail (MT) sites. **C, D** Immunohistochemistry (IHC) demonstrating abundant neutrophils, as stained by anti-MPO antibodies, present in SKG spine base enthesis (**C**) and at ankle enthesis (**D**). Black arrow: vertebral body. Red arrowhead: intervertebral disc. Black arrowhead: site of insertion of a tendon. Characteristic neutrophil trilobed nuclei can be seen in enlarged insets (bottom right)

SKG mice develop consistent inflammation in the axial spine and at peripheral sites, which occurs as early as 1–2 weeks post curdlan administration and can be quantified clinically (Fig. 1A). Histology of spine sections from the thoracic region to the mid tail were evaluated at 1, 2, 3, 6 and 11 weeks post curdlan administration (*n* = 3 per time point). H&E staining confirmed that an inflammatory infiltrate developed as early as 1–2 weeks post curdlan at entheseal sites in the spine base. There was a continuum of a decreasing inflammatory entheseal infiltrate at more proximal vertebral sites (Fig. 1B). Similarly, H&E staining also confirmed peripheral entheseal infiltrate at ankle sites as early as 1–2 weeks post curdlan administration. These findings are in keeping with prior visualization of inflammation at these sites through infrared in vivo imaging [33].

**Fig. 2:**
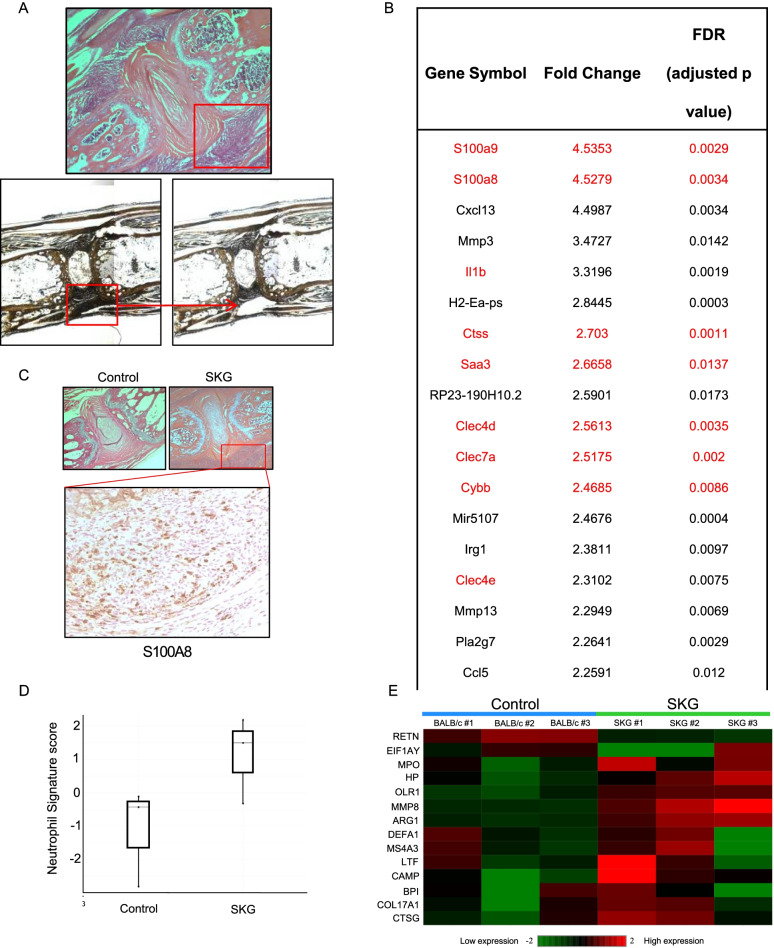
S100A8/A9 and other neutrophil-related genes are the most highly expressed at SKG spine enthesis. **A** Inflamed spine base enthesis (red square) microdissected via laser capture microscopy (H&E, upper panel) and in unstained paraffin sections before (left lower panel) and after (right lower panel) microdissection, ×4 magnification. **B** Top twenty most highly expressed genes in spine base entheses in Affymetrix gene array. S100A8 and S100A9, components of calprotectin, and other neutrophil-related genes are highlighted in red. **C** H&E stain of inflammatory infiltrate at axial spine enthesis in control BALB/c and SKG mice (red square), ×4 magnification, and IHC for S100A8 (×20 magnification) confirming protein expression of S100A8 at this site. **D, E** Murine neutrophil signature score (**D**) and heatmap (**E**) show that the neutrophil signature is significantly prominent in spine base inflamed enthesis from SKG compared to BALB/c control mice at entheseal sites (*n*= 3 per group). Granulocyte signature of resting granulocytes from human peripheral blood was used as a reference in **D** [31]

Multi-lobed nuclei of neutrophils were easily visualized in tissues at spine and ankle entheseal sites, and these cells stained positive for myeloperoxidase (MPO), a marker for neutrophils (Fig. 1C, D). Consistent with prior studies, gene array analysis from tissues at both ankle and spine inflamed entheseal sites revealed that regulators of pro-inflammatory cytokine genes including TNF, IL-1β, IL-6, IL-17, IL-23 and IL-12 were activated (Supplementary Table 2). In order to further evaluate the type of early cellular infiltration and gene expression patterns at spine and ankle entheseal sites, inflamed entheseal tissue from the axial skeleton and ankles of SKG and control BALB/c mice (*n* = 3 per group) was microdissected via laser capture microscopy 3 weeks post curdlan administration (Fig. 2A and Supplementary Figure 1).

### Neutrophil-associated genes and pathways are highly upregulated at axial and peripheral enthesis sites during early inflammation in SKG mice

The early inflamed entheseal tissue obtained by laser capture from SKG and BALB/c control spine entheseal sites (*n*= 3 per group) was subjected to Affymetrix gene array analysis. A cutoff of greater than 2-fold change in gene expression compared to control was deemed significant. The most highly upregulated genes at both axial (Fig. 2B) and peripheral entheseal sites (Supplementary Table 3) in SKG mice were S100A8 and S100A9, the protein products of which form the heterodimer alarmin calprotectin. This alarmin is highly expressed intracellularly by neutrophils and is released extracellularly upon their activation. Protein expression of S100A8 was confirmed through IHC (Fig. 2C). Activation of upstream regulators of MYD88 and TLR4 (the endogenous receptor for S100A8/A9 agonists) was also found, in line with previously published data highlighting these genes in S100A8/A9 signaling (Supplementary Table 2) [34, 35].

The strong presence of neutrophils within entheseal infiltrates was supported by the top twenty perturbed pathways in the signaling pathway impact analysis (SPIA) [30], where multiple pathways related to neutrophil function, survival and differentiation were activated, highlighted in red in Table 1. Furthermore, the presence of neutrophils was confirmed through the neutrophil gene signature scores and heat map (Fig. 2D and E) using a previously validated neutrophil gene signature [32]. In addition, a significant fold-change expression of several neutrophil-associated genes was noted in the top 20 expressed genes at spine axial enthesis, highlighted in red in Fig. 2B. Notable among these genes are C-type lectin domain family 7 member A (Clec7a, Dectin-1), typically expressed by myeloid cells (neutrophils, macrophages and dendritic cells) [36], Cathepsin S (Ctss), known to activate IL-36 and drive neutrophil biology in psoriasis [37], C-type lectin domain family 4 member D (Clec4d), implicated in the recruitment of neutrophils at sites of inflammation [38], and Cytochrome B-245 Beta Chain (Cybb), encoding a subunit of Nox2, an important gene in reactive oxygen species production by neutrophils [39, 40].

**Table 1:**
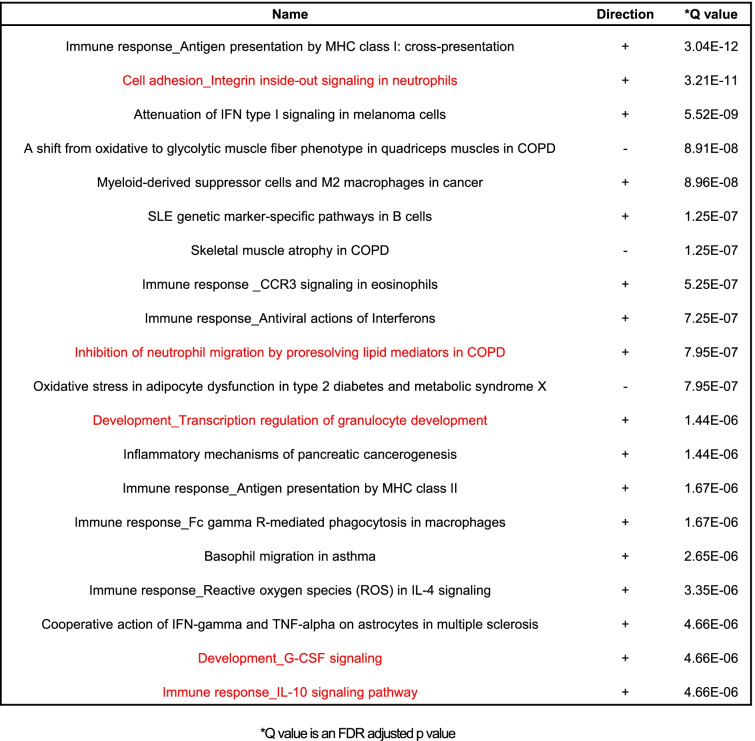
Activated pathways related to neutrophil function, survival and differentiation at axial enthesis from SKG mice

### Neutrophils are present at healthy human entheses and express IL-23 upon stimulation with fungal adjuvant

**Fig. 3:**
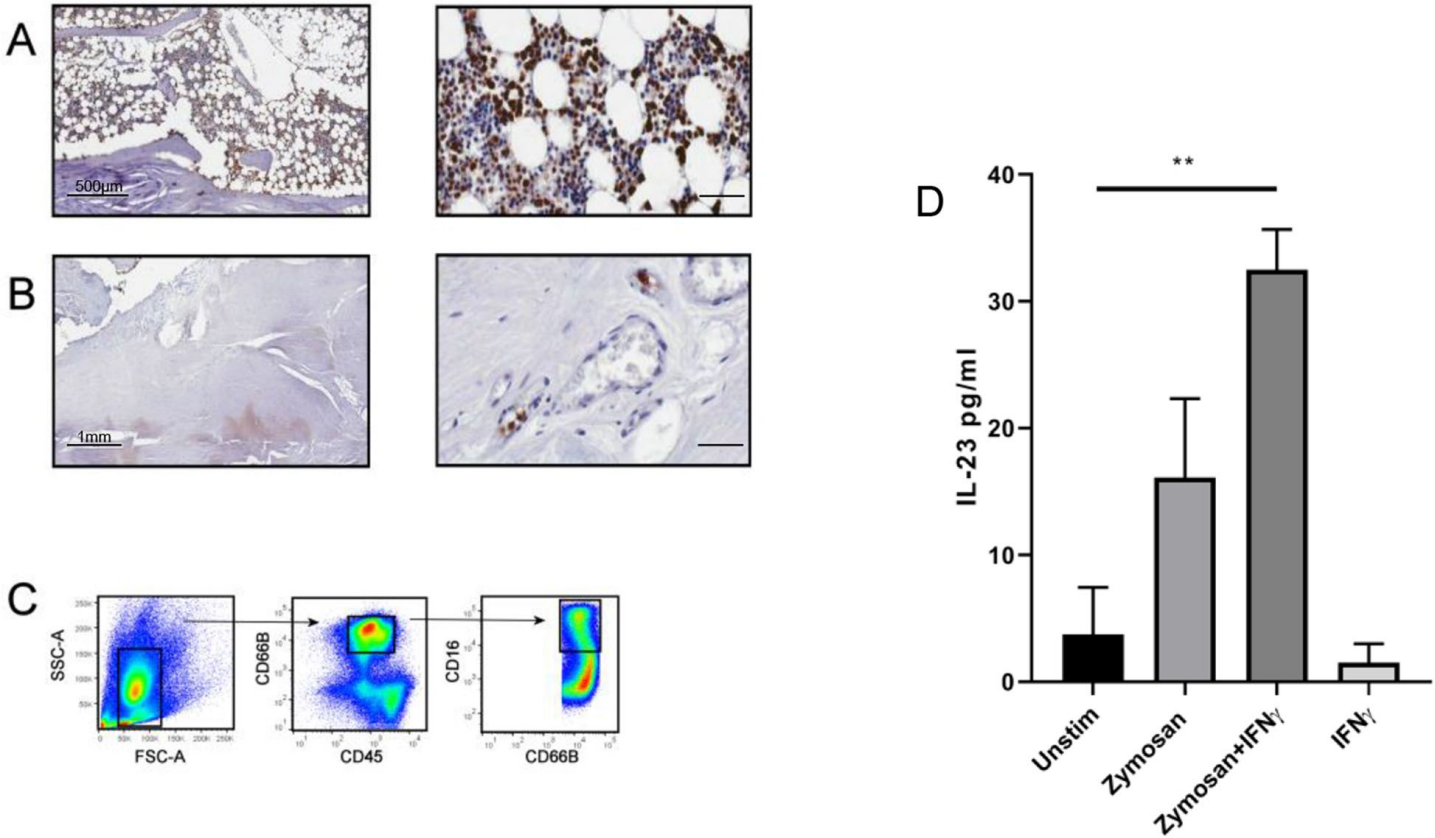
Neutrophils at healthy human axial enthesis are capable of producing IL-23 upon fungal adjuvant stimulation. **A** IHC for MPO, human non-inflamed axial enthesis: MPO-expressing neutrophils are present in peri-entheseal bone (3 samples examined). Scale bar (right) represents 100 μm. **B** MPO-expressing neutrophils are also present in entheseal soft tissue, where they are localized to the blood vessels and are seen marginating (3 examined). Scale bar (right) represents 100 μm. **C** Digestion of the peri-entheseal bone with subsequent cell sorting also shows that neutrophils are present at/near human axial enthesis (CD45, all leukocytes; CD66b, granulocyte marker; CD16, neutrophil specific marker) (*n* = 3 samples). **D** Axial entheseal neutrophils (*n* = 4 samples) were stimulated with the fungal adjuvant zymosan (0.5mg/ml), zymosan plus IFNγ (20ng/ml) and IFNγ alone for 48 h. IL-23 was measured by a sandwich ELISA for p40 and p19. Paired *t* test (**p < 0.01)

To confirm the presence of neutrophils in the human enthesis, tissue sections from human spinal enthesis samples (Supplementary Figure 2) were stained for MPO. MPO expressing cells were present in the peri-entheseal bone marrow (Fig. 3A) but were not detectable in the enthesis soft tissues. However, neutrophils showed margination in the blood vessels found within entheseal soft tissue (Fig. 3B), suggesting that these neutrophils are activated. Following enzymatic digestion of the non-inflamed human peri-entheseal bone and enthesis soft tissue, flow cytometry also confirmed the presence of neutrophils (CD45+ CD66b+ CD16+) (Fig. 3C) in peri-entheseal bone marrow but not in entheseal soft tissue (data not shown). It has previously been shown in mice that intraperitoneal fungal adjuvants rapidly spread to the joints and tibial bone marrow [41].

The SKG model is an IL-23-dependent model accelerated by fungal adjuvant that demonstrates arthritis and enthesitis, including early neutrophilic inflammation. Thus, we tested the ability of human entheseal neutrophils to secrete IL-23, as IL-23 secreting cells could initiate inflammation in SpA through activation at these sites by fungal and other adjuvants. When stimulated with the fungal adjuvant zymosan, enthesis-derived neutrophils significantly upregulated secretion of IL-23 (Fig. 3D), a finding enhanced by the co-administration of IFNγ, but not by administration of IFNγ alone. We also tested isolated blood neutrophils stimulated with two different fungal adjuvants (0.5mg/ml) for production of IL-23, as measured by ELISA (*n*=3). Mannan and zymosan (Supplementary Figure 3) show similar IL-23 induction capacity in neutrophils, while curdlan shows stronger induction capacity.

### Stromal cells from healthy human entheses produce proinflammatory chemokines upon fungal adjuvant administration

**Fig. 4:**
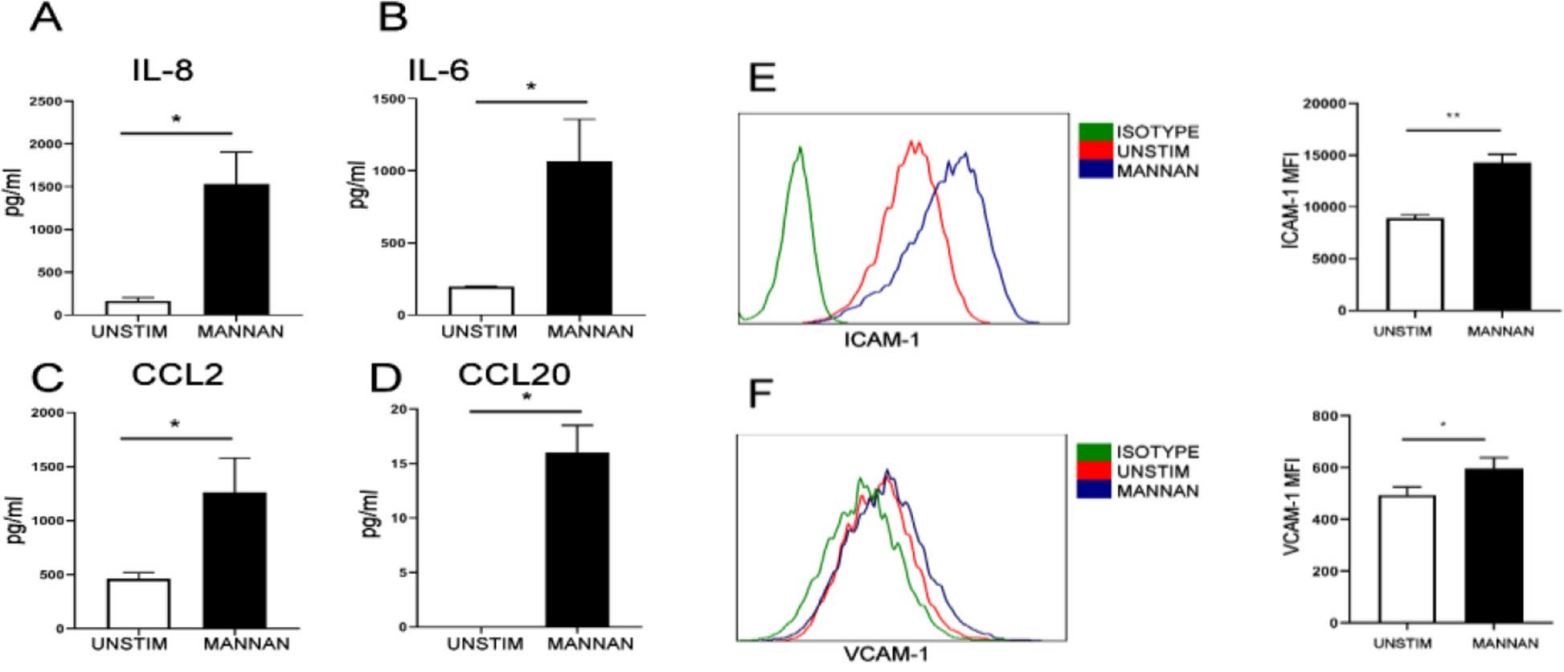
Human entheseal stromal cells produce chemokines and adhesion molecules important for leukocyte recruitment. Stromal cells were isolated from human entheseal sites and stimulated with the fungal adjuvant mannan (1 mg/ml) for 24 h. **A**–**D** Supernatant was analyzed for IL-8, IL-6, CCL2 and CCL20 protein (*n* = 3). **E**, **F** Expression of the adhesion molecules ICAM-1 and VCAM-1 was analyzed by FACS, and median fluorescence intensity was quantified (*n* = 3). Paired *t* test (*p < 0.05, **p < 0.01)

Activation of stromal cells from entheseal sites is thought to play a role in the initiation of enthesitis [42]. When stimulated with the fungal adjuvant mannan, entheseal stromal cells significantly upregulated secretion of the neutrophil chemoattractant IL-8 (Fig. 4A). Mannan stimulation also resulted in the significant upregulation of other disease-relevant chemokines and cytokines including IL-6 (involved in promoting the IL-23/IL-17 axis), CCL20 (chemoattractant for IL-17-expressing cells) and CCL2 (chemoattractant for monocytes) (Fig. 4B–D). Adhesion molecules, whose upregulation is required for leukocyte recruitment, were also upregulated by mannan stimulation, including ICAM-1 (Fig. 4E) and VCAM-1 (Fig. 4F).

## Discussion

We report that neutrophils are present in both non-inflamed human and diseased murine entheses. Since neutrophils are short-lived and thus difficult to manipulate and study in human samples, we have used the SKG mouse as a model of SpA. We confirm a key early role for neutrophils in the SKG enthesitis and for the first time confirm the presence of human enthesis neutrophils that have the capability to produce IL-23.

We demonstrate the presence of neutrophils by H&E staining and expression of MPO and by noting the presence of a neutrophil gene signature and upregulation of neutrophil-associated pathways amidst the 20 most activated pathways in IPA analysis. In the early entheseal inflammatory infiltrates in the spine, we find a highly upregulated gene and protein expression of the alarmins S100A8 and S100A9, components of calprotectin, a myeloid lineage factor. Calprotectin has been shown to be upregulated in the psoriatic skin and has been implicated in disease pathogenesis [43]. Calprotectin is also a biomarker for several inflammatory diseases, most notably inflammatory bowel disease, and is a marker of radiographic progression in SpA [44]. Furthermore, S100A8 and S100A9 are products of neutrophils and monocytes and are known to modulate the early stromal microenvironment during the development of tendinopathy and to influence the composition of the inflammatory cell infiltrate [45].

Our integrated evaluation of murine and human tissue supports an important role of neutrophils in the earliest stages of SpA. We report that neutrophils isolated from non-inflamed human entheses are capable of producing IL-23 in response to stimulation with the fungal adjuvant zymosan. Zymosan activates TRL2 and Dectin-1 pathways, and in line with our observations, it has been reported that IL-23 is also produced by peripheral blood neutrophils using TLR agonists [24]. In addition IL-23 producing CD14+ monocytes have been found in human entheses [46]. Until recently, IL-23 was thought to be largely produced by professional antigen presenting cells such as macrophages and dendritic cells. However, Kvedaraite et al. have reported that in IBD, the main source of IL-23 is tissue infiltrating neutrophils [22]. In our study, we also show that human entheseal stromal cells can produce chemokines associated with neutrophil chemotaxis following fungal adjuvant stimulation. It has been previously demonstrated that through an autocrine effect of S100A8/A9 on neutrophils, there is promotion of their slow-rolling and adhesion in the blood vessel walls [47]. Interestingly, in non-inflamed human entheseal tissue, we observe neutrophils lining blood vessels. We hypothesize that once present at entheseal sites, activated neutrophils could initially promote further neutrophil infiltration and contribute to the propagation of inflammation when normal tissue reparative processes are dysregulated. This hypothesis needs to be validated through additional work.

Limitations of this study include the fact that other myeloid cells may play a role in entheseal pathology in the SKG model and are likely contributors in early inflammation. We hypothesize that there is an upregulation of IL-8 in local fibroblasts, with subsequent recruitment of neutrophils, but we do not exclude a role for monocytes/macrophages or other cell types in early disease pathogenesis. Instead, we highlight the presence of neutrophils, an underappreciated cell in entheseal pathogenesis. We have not isolated neutrophils from SKG mice to directly show production of IL-23 upon fungal stimulation (as was done with human peri-entheseal bone neutrophils). However, we infer this association from the combination of gene array analysis and histologic data, where a prominent neutrophilic infiltrate is present at early timepoints in inflammation. Finally, we note that although IL-23 inhibition has been shown to be ineffective in treating axial SpA patients with established disease [48, 49], it is still likely that it plays a role as an important contributing cytokine in the development of enthesitis and possibly also as a key contributor in early SpA pathogenesis.

## Conclusion

The SpA group of diseases are strongly linked to the IL-23/17 cytokine axis, and are linked to neutrophil biology including maturation, marrow egression, tissue infiltration and activation. Furthermore, the target organs in SpA, including the skin, eye and gut, as well as more rare skeletal diseases including SAPHO, show significant macroscopic or microscopic neutrophil infiltration [39]. Our data suggest that neutrophils play an important role in the earliest enthesitis lesions in the SKG mouse model of SpA (an IL-23/17 dependent model), and we demonstrate that the normal human enthesis contains neutrophils that, when stimulated, can produce IL-23. These findings suggest that this innate immune cell type might play a key role in disease pathogenesis.

## Supplementary Information

**Additional file 1: Supplementary Table 1.** Antibodies used for the indicated application. FC: flow cytometry. IHC: immunohistochemistry. **Supplementary Table 2.** Genes for which upstream regulators were identified in murine axial enthesis. Z-scores obtained from IPA upstream regulator analysis show significant gene expression of upstream regulators of pro-inflammatory cytokines important in development of SKG arthritis including TNF, IL1B, IL6, IL17, IL23 and IL12, as well as MYD88 and TLR4, known mediators of S100A8/A9 signaling, all depicted in red. **Supplementary Table 3.** Most highly expressed genes in SKG ankle entheses in Affymetrix gene array. Shown in red are the two most highly expressed genes in SKG ankle enthesis (also in SKG axial enthesis), S100A8 and S100A9, products of myeloid cells including neutrophils. Shown in black are other highly expressed genes.

**Additional file 2: Supplementary Figure 1.** Left panel: H and E-stained histologic section of the ankle and midfoot. Tibia, talus and navicular bones are indicated. Right panel: Sites of tissue procured from laser capture microdissection. Upper panel shows sites prior to dissection (red circles); lower panels show sites after dissection (red arrows). **Supplementary Figure 2.** Illustration of human spine depicting Peri Entheseal bone and Entheseal Soft Tissue sites dissected and used for subsequent analysis in this study. **Supplementary Figure 3.** Isolated blood neutrophils were stimulated with different fungal adjuvants, mannan, zymosan and curdlan at 0.5mg/ml and IL-23 was measured by ELISA (n=3).

## Data Availability

The datasets used and/or analysed during the current study are available from the corresponding author on reasonable request.

## Abbreviations

SpA: Spondyloarthritis
MTA: Mouse transcriptome assay
IPA: Ingenuity pathway analysis
AS: Ankylosing spondylitis
ReA: Reactive arthritis
PsA: Psoriatic arthritis
IBD: Inflammatory bowel disease
SAPHO: Synovitis, acne, pustulosis, hyperostosis and osteitis
CRMO: Chronic recurrent multifocal osteomyelitis
ZAP-70: Zeta chain-associated protein kinase 70
CARD9: Caspase recruitment domain family member 9
LCM: Laser capture microdissection
H&E: Hematoxylin and eosin
FFPE: Formalin-fixed paraffin-embedded
SA-HRP: Streptavidin horseradish peroxidase
MPO: Myeloperoxidase
DAB: Diaminobenzidine
PBS: Phosphate-buffered saline
EDTA: Ethylene diamine tetra-acetic acid
FACS: Fluorescence-activated cell sorting
DAPI: 4′,6-diamidino-2-phenylindole
IFNγ: Interferon-gamma
SPIA: Signaling pathway impact analysis
GSVA: Gene set variation analysis
IHC: Immunohistochemistry
