# Supplementary figures and images for: A role for neutrophils in early enthesitis in spondyloarthritis
Source: Arthritis Res Ther. 2022 Jan 17;24:24. doi: 10.1186/s13075-021-02693-7 (PMC8762869; doi:10.1186/s13075-021-02693-7)

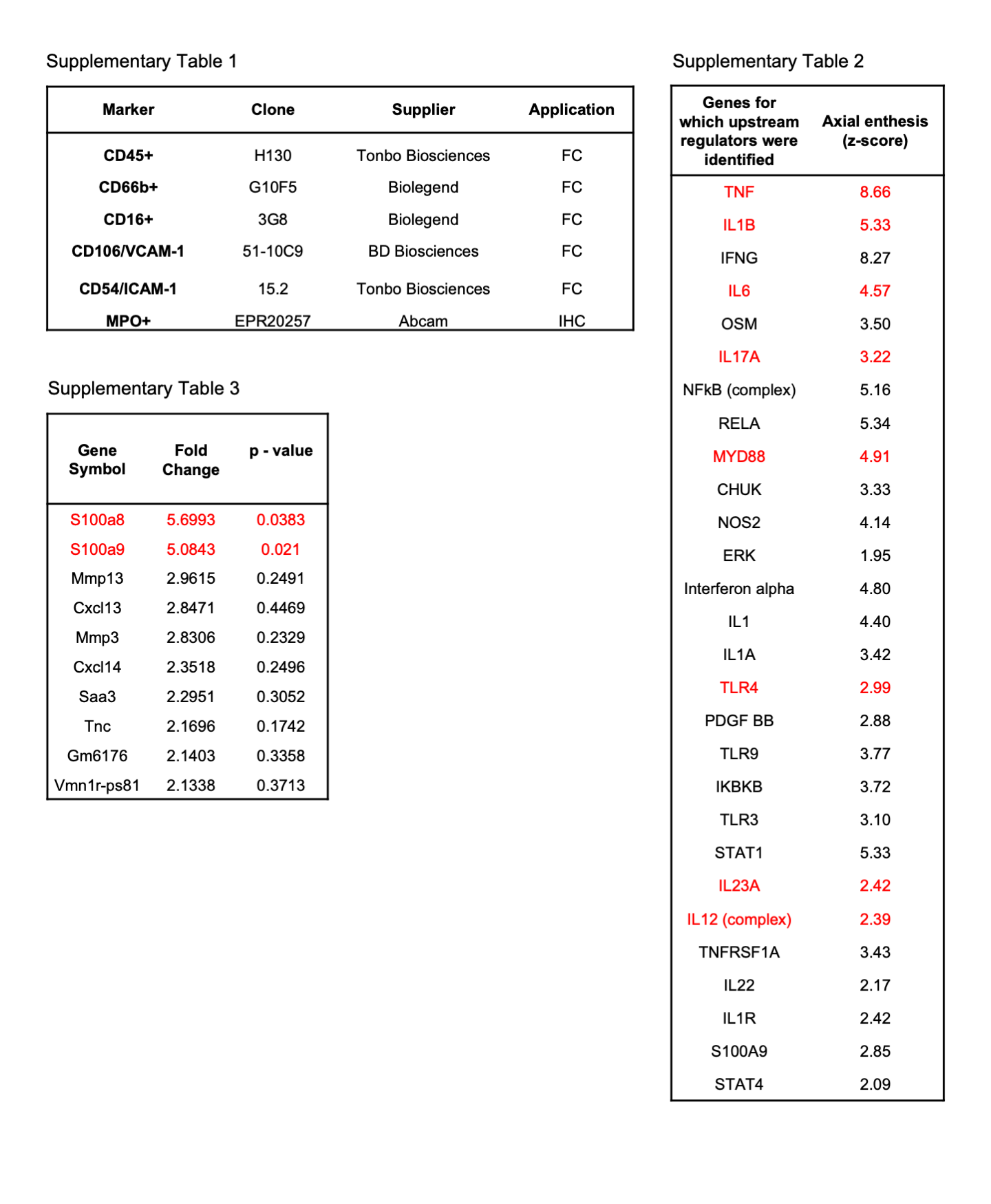

Supplement: Supplementary file 1 — Additional file 1: Supplementary Table 1. Antibodies used for the indicated application. FC: flow cytometry. IHC: immunohistochemistry. Supplementary Table 2. Genes for which upstream regulators were identified in murine axial enthesis. Z-scores obtained from IPA upstream regulator analysis show significant gene expression of upstream regulators of pro-inflammatory cytokines important in development of SKG arthritis including TNF, IL1B, IL6, IL17, IL23 and IL12, as well as MYD88 and TLR4, known mediators of S100A8/A9 signaling, all depicted in red. Supplementary Table 3. Most highly expressed genes in SKG ankle entheses in Affymetrix gene array. Shown in red are the two most highly expressed genes in SKG ankle enthesis (also in SKG axial enthesis), S100A8 and S100A9, products of myeloid cells including neutrophils. Shown in black are other highly expressed genes. [file 13075_2021_2693_MOESM1_ESM.tiff]

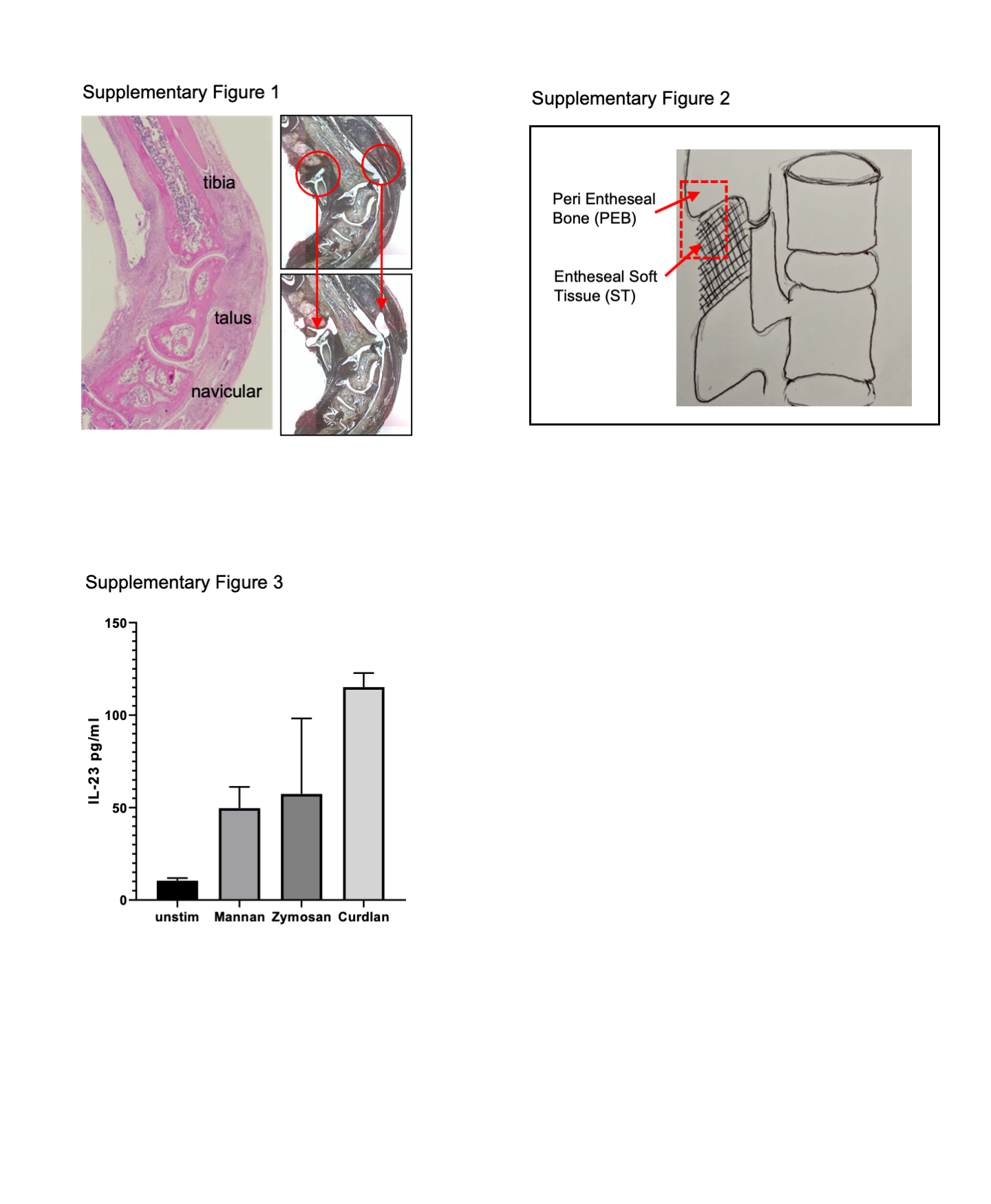

Supplement: Supplementary file 2 — Additional file 2: Supplementary Figure 1. Left panel: H and E-stained histologic section of the ankle and midfoot. Tibia, talus and navicular bones are indicated. Right panel: Sites of tissue procured from laser capture microdissection. Upper panel shows sites prior to dissection (red circles); lower panels show sites after dissection (red arrows). Supplementary Figure 2. Illustration of human spine depicting Peri Entheseal bone and Entheseal Soft Tissue sites dissected and used for subsequent analysis in this study. Supplementary Figure 3. Isolated blood neutrophils were stimulated with different fungal adjuvants, mannan, zymosan and curdlan at 0.5mg/ml and IL-23 was measured by ELISA (n=3). [file 13075_2021_2693_MOESM2_ESM.tiff]
